# Pathological Investigation of the Effect of Bovine Colostrum Against 5-FU-Induced Liver, Kidney, and Heart Toxicity in Rats

**DOI:** 10.3390/life15040564

**Published:** 2025-03-31

**Authors:** Muhammet Bahaeddin Dörtbudak, Muhammed Demircioğlu, İsmail Demircioğlu, Mario Nicotra, Alessandro Di Cerbo

**Affiliations:** 1 Department of Pathology, Faculty of Veterinary Medicine, Harran University, 63300 Sanliurfa, Turkey; 2 Department of Histology and Embryology, Institute of Health Sciences, Dicle University, 21280 Diyarbakir, Turkey; mdemircioglu63@hotmail.com; 3 Department of Anatomy, Faculty of Veterinary Medicine, Harran University, 63300 Sanliurfa, Turkey; idemircioglu@harran.edu.tr; 4 School of Biosciences and Veterinary Medicine, University of Camerino, 62024 Matelica, Italy; mario.nicotra@unicam.it

**Keywords:** cancer, 5-FU, hepatotoxicity, nephrotoxicity, cardiotoxicity, pathology

## Abstract

This study aimed to investigate the possible histopathological and immunohistochemical effects of bovine colostrum (BC) against the toxic effects of 5-fluorouracil (5-FU) on the liver, kidney, and heart of Wistar Albino rats. Animals were divided into three groups: control, 5-FU, and 5-FU+BC. The control group received 2 mL/kg i.p. saline, the 5-FU group 100 mg/kg i.p. 5-FU, and the 5-FU+BC group received 100 mg/kg i.p. saline on the first day of the study. The 5-FU and 5-FU+BC groups received 100 mg/kg i.p. of 5-FU and 1000 mg/kg BC orally each day of the study. Liver, kidney, and heart tissues were examined histopathologically for lesions and the expression of TNF-α, HSP-27, CASP-3, and 8-OHdG. No pathologic lesions were observed in the control group, whereas severe pathologic lesions were observed in the 5-FU group. In the 5-FU+BC group, the lesions were less severe than in the 5-FU group. In immunohistochemical examination, biomarker expression was not observed in the control group, whereas it was severe in the 5-FU group and less severe in the 5-FU+BC group. At the end of the study, it was observed that 5-FU-induced pathological findings in liver, kidney, and heart tissues decreased with the use of bovine colostrum. The difference between the control group and the 5-FU and 5-FU+BC groups was significant (*p* < 0.01 and *p* < 0.05, respectively). Although the BC addition did not show any statistical significance in the pathological scores of 5-FU in liver, kidney, and heart tissues, it was observed that it improved the lesions of these tissues. Nevertheless, histopathological and immunohistochemical analyses showed visible improvements in the 5-FU+BC group. Although more studies are needed, it is hoped that BC will improve prognosis by both reducing the side effects of 5-FU, a good chemotherapeutic agent, and its antineoplastic properties.

## 1. Introduction

Various chemotherapeutic agents are used in the treatment of cancer, one of the most critical health problems of today. Among these, 5-fluorouracil (5-FU) is used to treat solid tumors of many tissues and organs such as the breast, stomach and intestine, liver, pancreas, head and neck, esophagus, testis, cervix, and skin [1,2]. 5-FU, a fluorinated pyrimidine analog, is metabolized intracellularly to fluorodeoxyuridine monophosphate, fluorodeoxyuridine triphosphate, and fluoruridine triphosphate. These antimetabolites integrate into RNA and DNA, inhibit thymidylate synthase, and limit deoxythymidine monophosphate (dTMP) synthesis, resulting in DNA damage targeting cancer cells. DNA damage and the concomitant inhibition of protein synthesis result in the antioxidant system’s impairment and oxidative stress. Increased oxidation products induce proinflammatory cytokine expression, leading to inflammation. In addition, oxidative stress may contribute to DNA damage via ROS-mediated lipid peroxidation and NF-κB activation, creating a vicious cycle [3]. DNA damage also stimulates apoptosis through p53 activation [4,5,6]. These cytotoxic events, caused by 5-FU, occur in cancer cells and healthy cells, revealing the destructive side effects of this chemotherapeutic agent.

Since 5-FU is eliminated from the body primarily through the liver and partially through the kidneys, cytotoxicity in this organ is frequent. The cardiovascular system, sensitive to 5-FU-induced nitric oxide (NO) increase, is also highly exposed to toxicity. In the pathogenesis of liver, kidney, and heart tissue damage, which are the organs where 5-FU toxicity is most frequently observed, DNA damage, proinflammation cytokine increase, oxidative stress, and apoptosis play a role [1,7,8,9].

Colostrum is the milk secreted in the udder in the first days after birth to meet the basic needs of the offspring. Unlike milk, colostrum has a very high nutritional value, a thick, yellow-brownish appearance, and a salty-bitter taste. Colostrum, which contains most of the essential components in the blood in the postnatal period, provides an adaptation of the fetus, which is fed with blood during pregnancy, to the period of feeding with milk in the postnatal period [10,11]. The main components of colostrum are classified as nutrients (carbohydrates, proteins, fats, minerals, and vitamins), immune factors (immunoglobulin (Ig)A, G, M, D, E, lactoferrin, cytokines, β-lactoglobulin, α-lactoglobulin, polypeptides, oligosaccharides, lysozyme), and growth factors (insulin-like growth factor 1 and 2 (IGF-1, IGF-2), transforming growth factor-α and β (TGF-α, TGF-β), epidermal growth factor (EGF), vascular endothelial growth factor (VEGF), platelet-derived growth factor (PDGF)). Colostrum obtained from cows also contains many bioactive components that benefit the organism. In recent studies, antioxidant, antineoplastic, antimicrobial, antiviral, antifungal, anti-inflammatory, and immunomodulatory effects of cow colostrum have been revealed, and it is thought to provide proactive and therapeutic benefits in solving various health problems in terms of its properties [12,13,14].

In this study, the possible effect of cow colostrum against the severe side effects of 5-FU, one of the main cancer treatment agents, in liver, kidney, and heart tissues was examined histopathologically. In addition, considering the cytotoxic pathogenesis of 5-FU, current biomarkers of inflammation (tumor necrosis factor-α (TNF-α)), oxidative stress (heat shock protein-27 (HSP-27)), apoptosis (caspase3 (CASP-3)), and DNA (8-hydroxyguanosine (8-OHdG)) damage in liver, kidney, and heart tissues were examined immunopathologically.

## 2. Materials and Methods

### 2.1. Experimental Design

Eighteen male Wistar Albino rats aged 8–12 weeks and weighing ~250 g were used in the study. Animals were maintained at 22 °C in an environment controlled by a 12:12 h light–dark cycle, and feed and water were given ad libitum. Animals were randomly divided into three groups (6 rats each). This study was conducted with the approval (7 April 2024) of the Harran University Animal Experiments Local Ethics Committee (Protocol Number: 2024/004/08).

Non-fat, pasteurized, and freeze-dried Nutra Summa Pure Bovine Colostrum Powder (Phoenix, AZ, USA) was used to ensure minimal denaturation of BC, Ig, and other bioactive molecules. A 100 g bovine colostrum powder produced in the first 24 h of calving contained 48 g protein, 16 g fat, 25 g carbohydrate, and 15 g IgG. The approximate content of the major growth factors is as follows: IGF-1 (133 ng/mg powder), EGF (14 ng/mg powder), and TGFβ (8 pg/mg powder) [15]. The product was diluted with distilled water and administered to each animal by gavage as follows:

Control group (n = 6): Animals received 2 mL/kg saline i.p. for 10 days at the same time as the other groups;

5-FU group (n = 6): this group received a single dose of 100 mg/kg 5-FU intraperitoneally on the first day of the study;

5-FU + BC group (n = 6): 5-FU+BC group received a single intraperitoneal dose of 100 mg/kg of 5-FU on the first day of the study and 1000 mg/kg BC orally for 10 days starting from the same day [16].

### 2.2. Histopathologic Examination

After 10 days of experimentation, euthanasia was performed on all animals, and liver, kidney, and heart tissue samples were fixed in 10% buffered formaldehyde. The tissues were then washed in running tap water and subjected to routine tissue processing (dehydration, cleaning, impression). Paraffin blocks were then prepared from each tissue. From these paraffin blocks, 4 µm thick regular and adhesive slides were sectioned using a rotary microtome (Leıca RM 2125, Buccinasco, Italy). The tissue sections were subjected to xylol and alcohol series, deparaffinization, and rehydration procedures and then stained with hematoxylin and eosin, respectively. Finally, the sections were dried with alcohol, cleaned with xylol, dripped with Entallan, and covered with a coverslip. Two blinded investigators evaluated six different microscopic 20x magnification files for each sample. The preparations examined under the light microscope (Olympus BX51, Tokyo, Japan) were scored as absent (−), mild (+), moderate (++), and severe (+++) according to the severity of the pathological lesions [17].

### 2.3. Immunohistochemical Examination

Deparaffinization and rehydration of the sections taken on adhesive slides were performed. Tissue sections were washed with PBS and kept in 3% H_2_O_2_ for 10 min for endogenous enzyme inactivation. The tissues were subjected to boiling–cooling in citrate buffer 3 times for antigen retrieval and washed again with PBS. A protein block was dripped on the PAP pen-lined tissue sections to prevent non-specific antigen binding. The tissue sections were then incubated with TNF-α (sc-52746, Santa Cruz Biotechnology, Inc., Heidelberg, Germany), HSP-27 (sc-1049, Santa Cruz), CASP-3 (sc-56053, Santa Cruz Biotechnology, Inc., Heidelberg, Germany), and 8-OHdG (sc-66036, Santa Cruz Biotechnology, Inc., Heidelberg, Germany) primary antibodies for 16 h at +4 °C. The tissues were washed with PBS and incubated with a Biotinized secondary antibody. The sections were rewashed with PBS and incubated with Streptoavidin peroxidase. In the sections rewashed with PBS and 3,3′-Diaminobenzidine (DAB), chromogen was dropped to mark immunoreactions and washed with PBS for the last time. Immunoperoxidase-stained tissues were background-stained with Mayer’s hematoxylin. Finally, the tissue sections were dried with alcohol, cleaned with xylol, dripped with Entellan, and covered with a coverslip. Two blinded investigators evaluated six different microscopic 20x magnification files for each specimen. The preparations were examined under a light microscope (Olympus, BX53, Tokyo, Japan) and scored as absent (−), mild (+), moderate (++), and severe (+++) according to the severity of immunoreactions [18].

### 2.4. Statistical Analysis

Data were analyzed using GraphPad Prism 9 software (GraphPad Software, Inc., La Jolla, CA, USA) and presented as floating bars (min to max) with a line at the mean for the score and as the means ± standard deviation (SD) for its fold change. Differences among degenerative-necrotic lesions (DNLs), inflammatory cell infiltrates (ICIs), and vascular changes (VCs) for liver, kidney, and heart pathology scoring were assessed using a Kruskal–Wallis test followed by Dunn’s post hoc test. A * *p* < 0.05 was considered significant.

## 3. Results

### 3.1. Histopathologic Analysis

**Figure 1:**
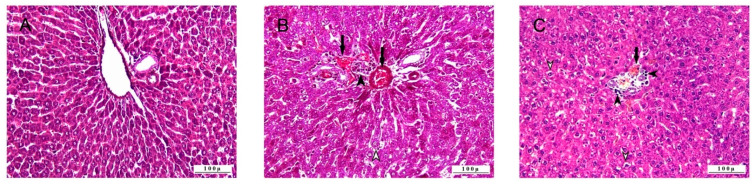
Histopathologic sections of liver (HE, X200): (**A**) control group with normal histological appearance, (**B**) 5-FU group with degenerative-necrotic lesions in hepatocytes (open arrowhead), inflammatory cell infiltrations (arrowhead), vascular changes (arrows), and (**C**) 5-FU+BC group with degenerative-necrotic lesions in hepatocytes (arrowheads), inflammatory cell infiltrations (arrowhead), and vascular changes (arrow).

Histopathologic examination of the liver tissues revealed that the control groups had no pathologic lesions and had a normal histologic appearance (Figure 1).

Severe pathologic changes were observed in 5-FU groups. Most liver hepatocytes showed degeneration (steatosis) with vacuolar character and partial intracytoplasmic localization of fat vesicles. Coagulation necrosis was observed in the central zone of hepatic lobules, with intense degenerative changes. In the parenchymal tissue, where degenerative and necrotic lesions developed, hepatocyte arrays were disrupted (dissociation), and dilatation occurred in the sinusoidal spaces. In the portal lobules, where severe cell damage was observed, vascular changes related to hyperemia and occasional hemorrhage were noted. Leukocyte cell infiltrations, mostly mononuclear type, were observed around the extremely hyperemic vessels in the portal lobe and the sinusoidal spaces. Pathologic findings were also observed in the 5-FU+BC group; however, the pathologic lesions in this group were not as severe as in the 5-FU group. Although intense degenerative lesions were observed in hepatocytes, necrosis was less, and the disruption in a radial arrangement in hepatocytes was not too much. There was severe hyperemia in the vessels, but hemorrhages were less common. The inflammatory cell infiltrations accompanying these vascular changes were not as intense as in the 5-FU groups.

**Figure 2:**
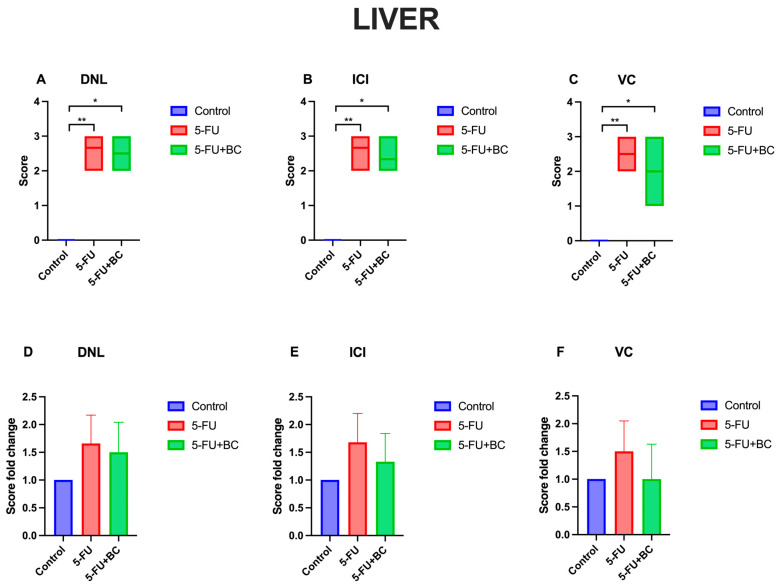
Graphical representation of liver pathology scoring for (**A**) DNL, (**B**) ICI, (**C**) VC, and (**D**–**F**) their relative fold change. * *p* < 0.05, ** *p* < 0.01. DNL (degenerative-necrotic lesion), ICI (inflammatory cell infiltrate), VC (vascular change).

The results concerning the liver pathology scoring analysis are summarized in Figure 2.

Compared to the control, 5-FU administration caused a significant increase (*p* < 0.01) in the score of all parameters (from a value of 0 to 2.66 ± 0.51 for DNL (1.66-fold increase), 2.67 ± 0.52 for ICI (1.66-fold increase), and 2.5 ± 0.54 for VC (1.50-fold increase)). A similar trend, but with a low significance (*p* < 0.05), was observed for the 5-FU+BC group compared to the control (from a value of 0 to 2.5 ± 0.55 for DNL (1.50-fold increase), to 2.33 ± 0.51 for ICI (1.33-fold increase), to 2.0 ± 0.63 for VC (1.0-fold increase)). Although not significant, adding BC ameliorated the toxic effect of 5-FU.

**Figure 3:**
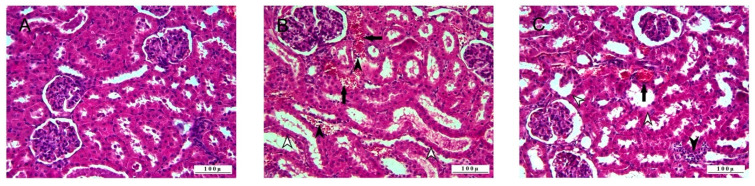
Histopathologic sections of kidney (HE, X200): (**A**) control group with normal histological appearance, (**B**) 5-FU group with degenerative-necrotic lesions in tubular epithelium (open arrowheads), inflammatory cell infiltrations (arrowheads), vascular changes (arrows), and (**C**) 5-FU+BC group with degenerative-necrotic lesions in tubular epithelium (open arrowheads), inflammatory cell infiltrates (arrowheads), and vascular changes (arrow).

Histopathologic examination of the kidney tissues revealed that the control group had no pathologic lesions and a normal histologic appearance (Figure 3).

In the 5-FU group, severe pathologic findings were observed in the kidneys. Severe degenerations in the tubule epithelium, mostly hydropic, were observed. Severe degenerative changes were accompanied by necrosis, and necrotic cells desquamated in tubule lumens. In addition, hyaline cylinders were observed in the tubule lumens, where distortion and cystic dilatation were observed.

The vessels were hyperemic, and severe hemorrhages were observed in the interstitial area. Leukocyte cell infiltrations were observed in the interstitial area where vascular changes were observed. Degenerative and necrotic lesions were also observed in the glomerulus cells, and the expansion of Bowman’s capsules due to sero-fibrinous exudation and atrophies in some glomeruli were observed.

Pathologic findings were observed in the 5-FU+BC groups, but pathologic lesions in this group were not as severe as in the 5-FU group. Severe degenerations were observed in tubule epithelial cells, but desquamated cells and hyalinized formations were rarely observed. Hyperemia was observed in the vessels in the interstitial area, but hemorrhage and leukocyte cell infiltrations were less in the 5-FU group.

**Figure 4:**
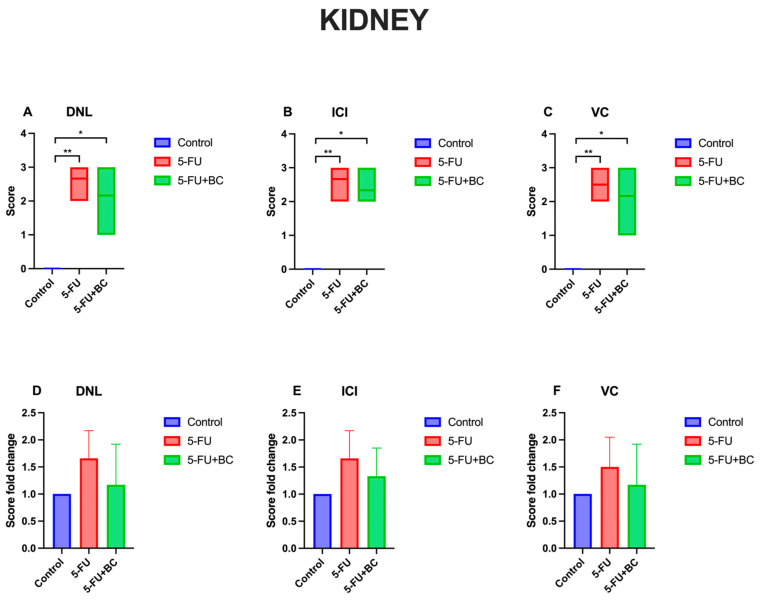
Graphical representation of kidney pathology scoring for (**A**) DNL, (**B**) ICI, (**C**) VC, and (**D**–**F**) their relative fold change. * *p* < 0.05, ** *p* < 0.01.

The results concerning the kidney pathology scoring analysis are summarized in Figure 4. 

Compared to the control, 5-FU administration caused a significant increase (*p* < 0.01) in the score and its fold change of all parameters, which were identical to that observed in the liver. On the other hand, despite a similar trend and significance (*p* < 0.05) of the 5-FU+BC group to that observed in the liver, the values were slightly different (from a value of 0 to 2.16 ± 0.75 for DNL (1.17-fold increase), to 2.33 ± 0.52 for ICI (1.33-fold increase), to 2.16 ± 0.75 for VC (1.17-fold increase)). Although not significant, adding BC ameliorated the toxic effect of 5-FU.

**Figure 5:**
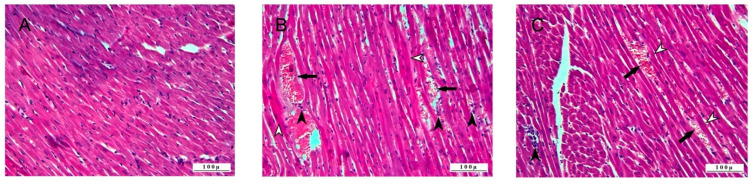
Histopathologic sections of heart (HE, X200): (**A**) control group with normal histological appearance; (**B**) 5-FU group, with degenerative-necrotic lesions in cardiomyocytes (open arrowheads), inflammatory cell infiltrates (arrowheads), vascular changes (arrows), and (**C**) 5-FU+BC group with degenerative-necrotic lesions in cardiomyocytes (open arrowheads), inflammatory cell infiltrates (arrowheads), and vascular changes (arrow).

The histopathologic examination of the heart tissue noted that the control group did not have pathologic lesions and had a normal histologic appearance (Figure 5).

Severe pathologic findings were observed in the heart tissues of the 5-FU group. Myofibrillar degeneration due to severe degenerative and necrotic lesions was observed in cardiomyocytes. Edema-induced dilatation of the intermuscular spaces was observed. Hemorrhage and leukocyte cell infiltrations with edema were observed in the intermuscular spaces. In addition, inflammatory cell infiltrations were also observed around the hyperemic vessels. Pathologic findings were observed in the 5-FU+BC group, but the pathologic lesions in this group were not as severe as in the 5-FU group.

Cardiomyocytes showed intense degenerative changes, but necrosis, loss of myofibrillar striation, and intermuscular dilatation were less. Vessels were hyperemic with less hemorrhage and leukocyte cell infiltration in the intermuscular spaces.

**Figure 6:**
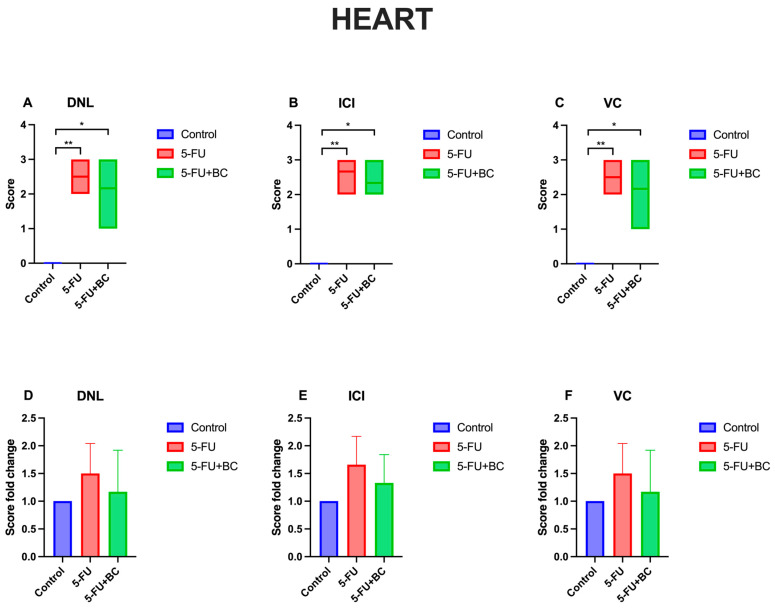
Graphical representation of heart pathology scoring for (**A**) DNL, (**B**) ICI, (**C**) VC, and (**D**–**F**) their relative fold change. * *p* < 0.05, ** *p* < 0.01.

The results concerning the heart function biomarkers’ analysis are summarized in Figure 6.

Also, for heart pathology scoring, 5-FU administration caused a significant increase (*p* < 0.01) in the score of all parameters compared to the control (from a value of 0 to 2.5 ± 0.54 for DNL (1.50-fold increase), to 2.66 ± 0.51 for ICI (1.66-fold increase), and 2.5 ± 0.55 for VC (1.50-fold increase)). On the other hand, the trend, significance (*p* < 0.05), values, and fold change were identical to those observed in the kidney. Although the pathological scoring was not statistically significant, the addition of BC was observed to improve 5-FU-induced histopathological lesions in the tissues.

### 3.2. Immunohistochemical Analysis

**Figure 7:**
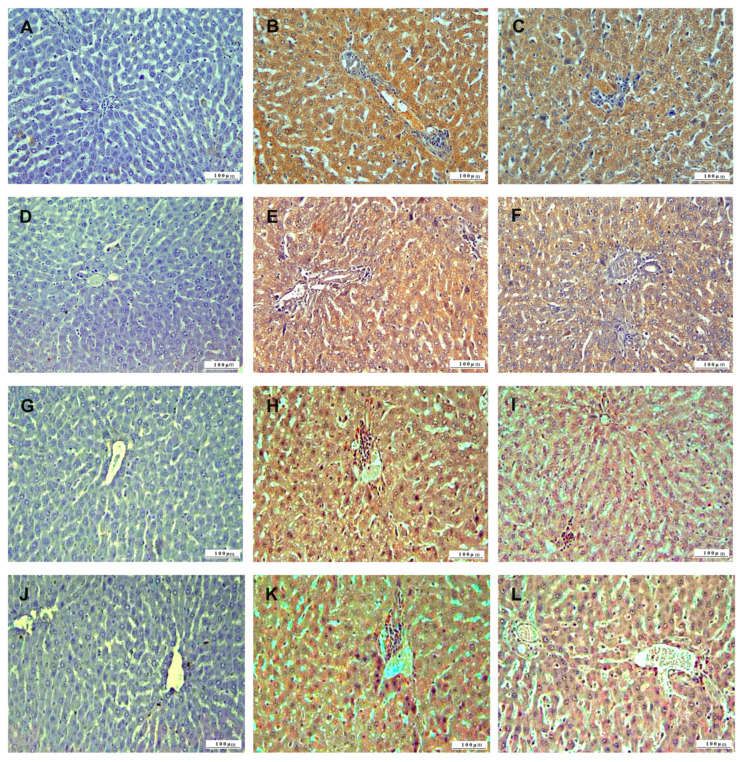
Immunohistochemical sections of the liver (IHC, X200): (**A**) control group TNF-α immunonegative; (**B**) 5-FU group with severe TNF-α expression, (**C**) 5-FU+BC group with TNF-α expression, (**D**) control group HSP-27 immunonegative (**E**) 5-FU group with severe HSP-27 expression, (**F**) 5-FU+BC group with HSP27 expression, (**G**) control group CASP-3 immunonegative, (**H**) 5-FU group with severe CASP-3 expression, (**I**) 5-FU+BC group with CASP-3 expression, (**J**) control group 8-OHdG immunonegative, (**K**) 5-FU group with severe 8-OHdG expression, and (**L**) 5-FU+BC group with 8-OHdG expression.

In the immunohistochemical examination of liver tissue, inflammation was assessed using TNF-α, oxidative stress using HSP-27, apoptosis using CASP-3, and DNA damage using 8-OHdG biomarkers (Figure 7). IL-2, CASP-3, HSP-27, and 8-OHdG expressions were not observed in the liver tissues of control group animals. In 5-FU group liver tissues, TNF-α, HSP-27, CASP-3, and 8-OHdG expressions were severe. In the 5-FU+BC group, liver tissue sections, TNF-α, HSP-27, CASP-3, and 8-OHdG expressions were less intense than in the 5-FU group.

**Figure 8:**
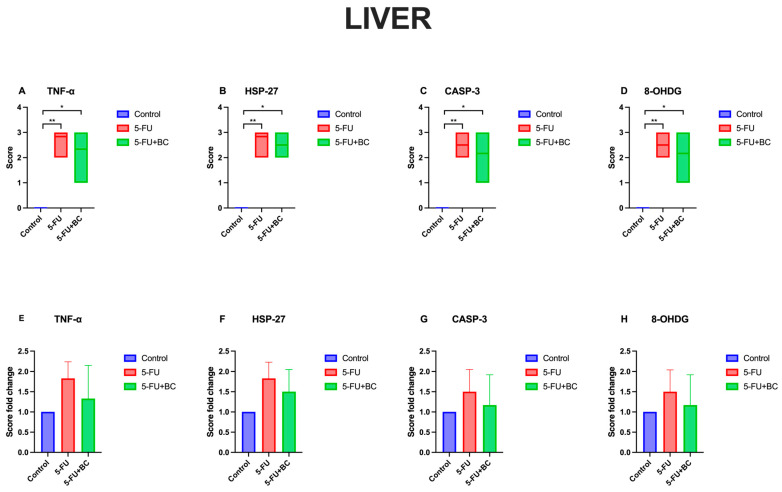
Graphical representation of (**A**) TNF-α, (**B**) HSP-27, (**C**) CASP-3, (**D**) 8-OHdG liver pathology scoring, and (**E**–**H**) their relative fold change. * *p* < 0.05, ** *p* < 0.01.

The results concerning the liver pathology scoring analysis are summarized in Figure 8.

Compared to the control, 5-FU administration caused a significant increase (*p* < 0.01) in the score of all parameters (from a value of 0 to 2.83 ± 0.41 for TNF-α (1.83-fold increase), to 2.83 ± 0.40 for HSP-27 (1.83-fold increase), to 2.5 ± 0.55 for CASP-3 (1.50-fold increase), to 2.17 ± 0.75 for 8-OHdG (1.50-fold increase)). A similar trend, but with a low significance (*p* < 0.05), was observed for the 5-FU+BC group compared to the control (from a value of 0 to 2.33 ± 0.81 for TNF-α (1.33-fold increase), to 2.5 ± 0.54 for HSP-27 (1.50-fold increase), to 2.16 ± 0.75 for CASP-3 (1.17-fold increase), to 2.16 ± 0.75 for 8-OHdG (1.17-fold increase)). Although the statistical analysis results obtained from the liver tissue were not significant, it was observed that the biomarker expressions related to 5-FU toxicity decreased with the addition of BC.

**Figure 9:**
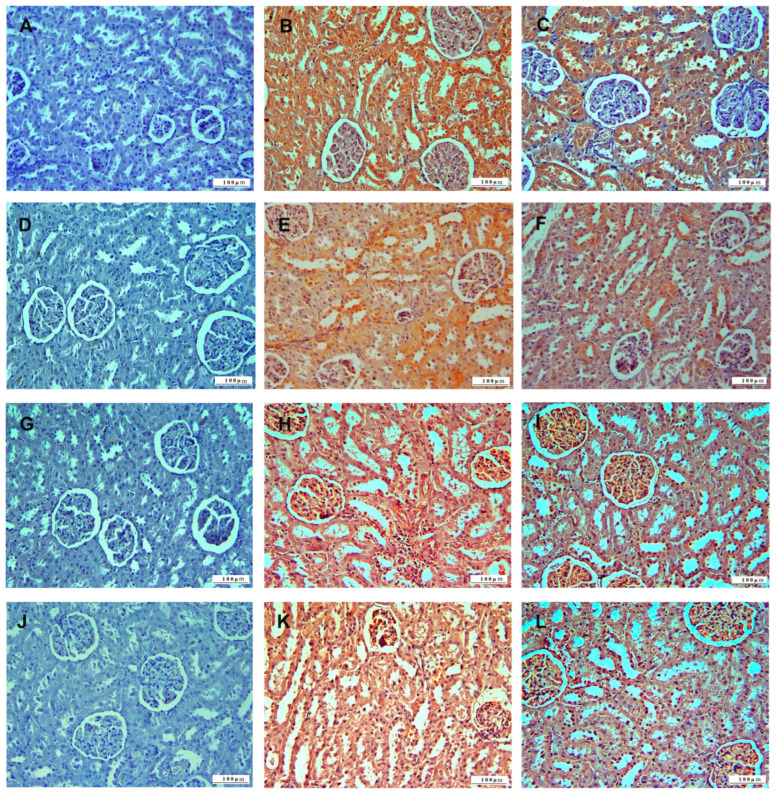
Immunohistochemical sections of the kidney (IHC, X200): (**A**) control group TNF-α immunonegative; (**B**) 5-FU group with severe TNF-α expression, (**C**) 5-FU + BC group with TNF-α expression, (**D**) control group HSP-27 immunonegative, (**E**) 5-FU group with severe HSP-27 expression, (**F**) 5-FU+BC group with HSP27 expression, (**G**) control group CASP-3 immunonegative, (**H**) 5-FU group with severe CASP-3 expression, (**I**) 5-FU+BC group with CASP-3 expression, (**J**) control group 8-OHdG immunonegative, (**K**) 5-FU group with severe 8-OHdG expression, and (**L**) 5-FU+BC group with 8-OHdG expression.

In the immunohistochemical examination of kidney tissue, TNF-α was used to monitor inflammation, as well as oxidative stress using HSP-27, apoptosis using CASP-3, and DNA damage using 8-OHdG biomarkers (Figure 9). TNF-α, HSP-27, CASP-3, and 8-OHdG expressions were not observed in the kidney tissues of the control group animals. TNF-α, HSP-27, CASP-3, and 8-OHdG expressions were observed in the kidney tissues of the 5-FU group. In the 5-FU+BC group’s kidney tissue sections, TNF-α, HSP-27, CASP-3, and 8-OHdG expressions were less intense than in the 5-FU group.

**Figure 10:**
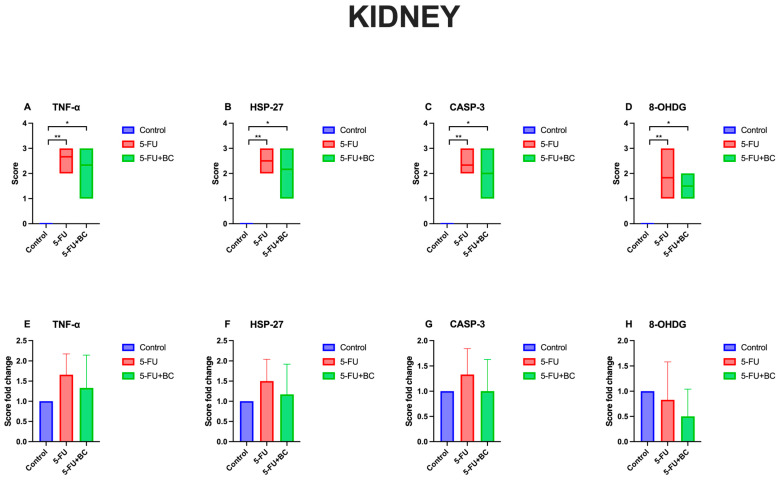
Graphical representation of (**A**) TNF-α, (**B**) HSP-27, (**C**) CASP-3, (**D**) 8-OHdG kidney pathology scoring, and (**E**–**H**) their relative score. * *p* < 0.05, ** *p* < 0.01.

The results concerning the kidney pathology scoring analysis are summarized in Figure 10.

As observed for the liver, 5-FU administration caused a significant increase (*p* < 0.01) in the score of all parameters compared to the control (from a value of 0 to 2.66 ± 0.51 for TNF-α (1.66-fold increase), to 2.5 ± 0.54 for HSP-27 (1.50-fold increase), to 2.33 ± 0.51 for CASP-3 (1.33-fold increase), to 1.83 ± 0.75 for 8-OHdG (0.83-fold increase)). A similar trend, but with a low significance (*p* < 0.05), was observed for the 5-FU+BC group compared to the control (from a value of 0 to 2.33 ± 0.81 for TNF-α (1.33-fold increase), to 2.16 ± 0.75 for HSP-27 (1.17-fold increase), to 2.0 ± 0.63 for CASP-3 (1.0-fold increase), to 1.5 ± 0.55 for 8-OHdG (0.50-fold increase)). Although not significant, adding BC ameliorated the toxic effect of 5-FU. Although the results obtained from the statistical analysis of the kidney tissue were not significant, it was observed that the biomarker expressions related to 5-FU toxicity decreased with the addition of BC.

**Figure 11:**
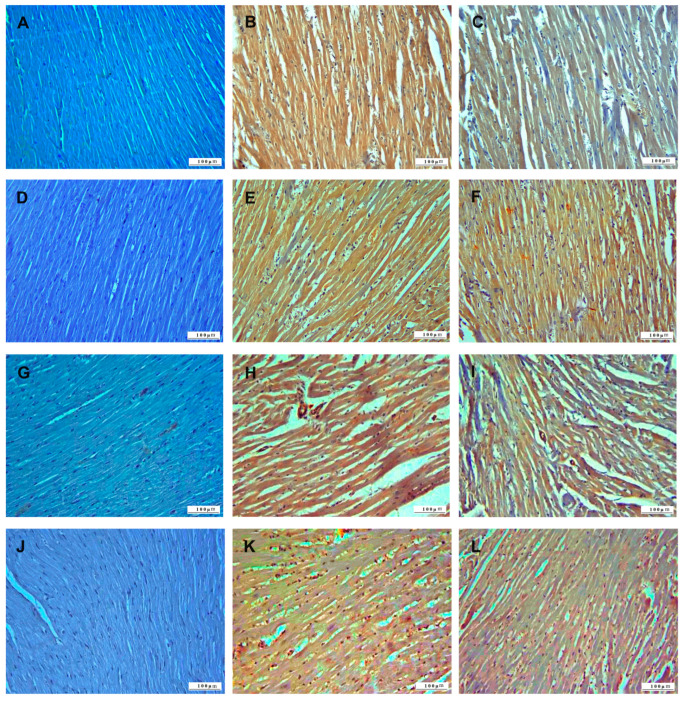
Immunohistochemical sections of the heart (IHC, X200): (**A**) control group TNF-α immunonegative; (**B**) 5-FU group with severe TNF-α expression, (**C**) 5-FU+BC group with TNF-α expression, (**D**) control group HSP-27 immunonegative, (**E**) 5-FU group with severe HSP-27 expression, (**F**) 5-FU+BC group with HSP27 expression, (**G**) control group CASP-3 immunonegative, (**H**) 5-FU group with severe CASP-3 expression, (**I**) 5-FU+BC group with CASP-3 expression, (**J**) control group 8-OHdG immunonegative, (**K**) 5-FU group with severe 8-OHdG expression, and (**L**) 5-FU+BC group with 8-OHdG expression.

In the immunohistochemical examination of heart tissue, TNF-α was used to monitor inflammation, as well as oxidative stress using HSP-27, apoptosis using CASP-3, and DNA damage using 8-OHdG biomarkers (Figure 11). TNF-α, HSP-27, CASP-3, and 8-OHdG expressions were not observed in the heart tissues of the control group animals. In the 5-FU group, heart tissues, TNF-α, HSP-27, CASP-3, and 8-OHdG expressions were severe. In the kidney tissue sections of the 5-FU+BC group, TNF-α, HSP-27, CASP-3, and 8-OHdG expressions were less intense than in the 5-FU group.

**Figure 12:**
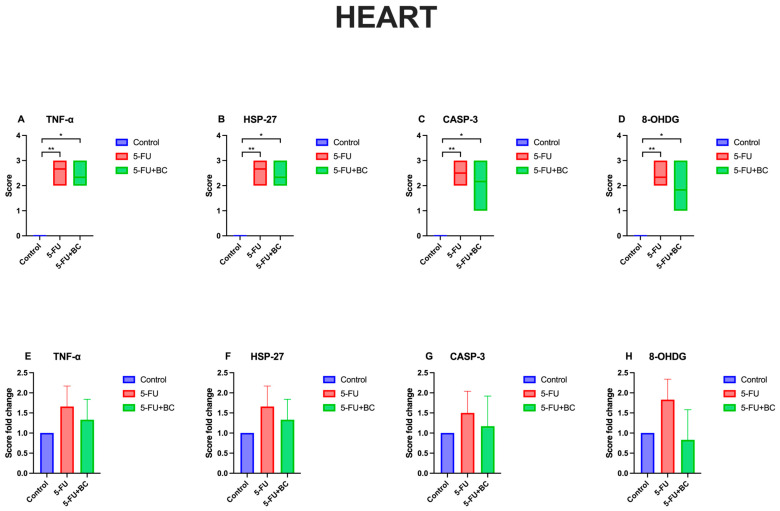
Graphical representation of (**A**) TNF-α, (**B**) HSP-27, (**C**) CASP-3, (**D**) 8-OHdG heart pathology scoring, and (**E**–**H**) their relative fold change. * *p* < 0.05, ** *p* < 0.01.

The results concerning the heart pathology scoring analysis are summarized in Figure 12.

As observed for the liver and the kidney, 5-FU administration caused a significant increase (*p* < 0.01) in the score of all parameters compared to the control (from a value of 0 to 2.67 ± 0.52 for TNF-α (1.66-fold increase), to 2.66 ± 0.52 for HSP-27 (1.66-fold increase), to 2.5 ± 0.54 for CASP-3 (1.50-fold increase), to 33 ± 0.75 for 8-OHdG (1.83-fold increase)). A similar trend, but with a low significance (*p* < 0.05), was observed for the 5-FU+BC group compared to the control (from a value of 0 to 2.33 ± 0.52 for TNF-α (1.33-fold increase), to 2.33 ± 0.51 for HSP-27 (1.33-fold increase), to 2.17 ± 0.75 for CASP-3 (1.17-fold increase), to 1.83 ± 0.75 for 8-OHdG (0.83-fold increase)). Although statistical analysis in heart tissue was not significant, it was observed that the biomarker expressions related to 5-FU toxicity decreased with the addition of BC.

## 4. Discussion

Approximately 10 million people die every year due to cancer, which is one of the leading global causes of death in our age. Chemotherapeutic agents are primarily used in the treatment of this life-threatening disease, and 5-FU is highly preferred. 5-FU is used in the treatment of many tissue and organ tumors. Like most chemotherapeutic agents, the working principle of 5-FU is its cytotoxic effect. The fact that the targets of chemotherapeutic agents are not only cancer cells but also healthy ones reveals their serious side effects, and this is a major concern for clinicians [1,19]. For this reason, minimizing or eliminating the striking side effects of chemotherapeutic agents with effective results in the fight against cancer has become the focus of the scientific world. Considering the pathogenesis of the cytotoxic mechanism in the fight against current side effects, antioxidant and anti-inflammatory products are thought to contribute to the solution. This study examined the possible impact of bovine colostrum, which has antioxidant, antineoplastic, antimicrobial, anti-inflammatory, and immunomodulatory properties due to the various bioactive factors it contains against 5-FU-induced organ toxicities [4,11,13].

DNA damage is the basis of 5-FU cytotoxicity. After a series of biological reactions that develop with DNA damage, an increase in oxidative stress, apoptosis, and inflammation occurs. 5-FU, which acts with the same pathogenesis regardless of healthy cells, creates toxic effects in many organs.

Approximately 80% of 5-FU applied for therapeutic purposes is inactivated in the liver, resulting in hepatotoxicity. Since 5-FU-induced hepatotoxicity negatively affects cancer prognosis, various studies have been conducted to reduce this side effect [1,20]. For instance, Yahya and Al-Shawi (2024) reported that nobiletin reduced caspase-mediated apoptosis, reducing 5-FU-induced hepatotoxicity and improving histopathological lesions [21]. Also, research conducted by Mansoori et al. (2024) proved that melatonin can reduce 5-FU-induced hepatotoxicity by suppressing oxidative stress and apoptosis [22]. A similar but less powerful effect was also achieved after the administration of cyclophosphamide. However, this molecule did not completely restore 5-fu-induced hepatotoxicity, reducing oxidative stress and resulting in organ damage [23]. Furthermore, 5-FU administration has been associated with increased inflammation (TNF-α, IL-1β) and oxidative stress (Keap-1, HO-1) biomarkers [24] and in the expressions of liver enzymes and some proinflammatory cytokines and apoptosis biomarkers [25]. In this sense, research conducted by Alharbi et al. (2024) proved that saroglitazar administration reversed the 5-FU toxic effect by regulating the interaction network of NF-kB and Nrf2 signaling pathways and apoptosis [24], while Sravathi et al. (2024) [25] showed that naringenin led to a restoration of the analyzed hepatic biochemical parameters, proving its hepatoprotective effect.

In the present study, 5-FU was observed to cause severe liver tissue histopathological lesions, inflammation, oxidative stress, apoptosis, and DNA damage. Nevertheless, BC application reduced histological destruction, inflammation, oxidative stress, apoptosis, and DNA damage, although the statistical analysis of the score did not reveal any significance.

Additionally, the kidneys eliminate about 15% of 5-FU from the body. It is broken down into α-fluoro-b-alanine, ammonia, urea, and carbon dioxide in the liver and catabolized as dihydrouracil. These metabolites cause nephrotoxicity with a similar cytotoxic effect in many organs. For this reason, various studies have been conducted to reduce this side effect [8,26].

For instance, Elbanan et al. (2023) reported that 5-FU caused histological destruction in kidney tissue and increased some proinflammatory and oxidative stress parameters, which, however, were ameliorated by melatonin administration, suggesting a renoprotective effect [27].

Also, chrysin, hesperidin, and curcumin have been shown to hold nephroprotective properties against 5-FU nephrotoxicity. In 2014, Rashid et al. reported that chrysin reduced the 5-FU-induced expression of apoptosis biomarkers such as p53, caspase-3, and Bax, thus reducing oxidative damage and apoptosis induced by this chemotherapeutic agent [28]. Hesperidin and curcumin reversed 5-FU-caused lipid peroxidation, apoptosis (CASP-3), and DNA damage (8-OHdG) in kidney tissue, with an overall nephroprotective effect [29]. The regulation of apoptosis is also the mechanism behind the nephroprotective effect of sinapic acid. In this regard, Ansari et al. showed that sinapic acid can regulate the expression of NF-κB and caspase-mediated proinflammatory cytokines, inhibiting 5-FU-induced renal apoptosis [30].

The present study observed that 5-FU caused severe histopathological lesions in kidney tissue, inflammation, oxidative stress, apoptosis, and DNA damage. Nonetheless, BC application reduced histological destruction and inflammation, oxidative stress, apoptosis, and DNA damage, although the statistical analysis of the score did not reveal any significance.

One of the most important complications of 5-FU is cardiotoxicity, where neuregulin 1 (NRG-1)/ErbB signaling impairment can lead to degeneration in myofibrils [31]. NO, which increases with the use of 5-FU, is the cause of damage to the endocardium and vascular endothelium [32]. Hence, various studies have been conducted to reduce the cardiotoxic side effects of 5-FU [5,33]. In 2022, Safarpour et al. showed that colchicine can counteract the onset of severe histological lesions in heart tissue and decrease the 5-FU-caused expression of some cardiac enzymes and inflammation biomarkers, resulting in a cardioprotective effect [34]. Also, bosentan was shown to reduce 5-FU-induced cardiotoxicity by modulating TLR4/MyD88/NFκB signaling pathways, thus determining antioxidant, anti-inflammatory, and antiapoptotic effects in cardiac tissue [34], as well as quercetin to suppress 5-FU-induced cardiac apoptosis (CASP-3), inflammation (TNF-α, IL-1β), and oxidative stress (NO) [35]. Similar effects were also assessed for thymoquinone and hesperidin using molecular docking [36] and simvastatin, whose activity was ascribed to activating the ROCK/Akt/eNOS and ET-1/ERK pathways [37].

The present study showed that 5-FU caused severe histopathological lesions in heart tissue, inflammation, oxidative stress, apoptosis, and DNA damage. However, BC application reduced histological destruction, inflammation, oxidative stress, apoptosis, and DNA damage, although the statistical analysis of the score did not reveal any significance.

Bovine colostrum, secreted from the mammary glands of cows after parturition and containing almost all the elements in blood, contains over 90 bioactive components beneficial to the organism. Previous studies on colostrum, which has a very high nutritional value, have reported its antioxidant, antineoplastic, antimicrobial, antiviral, antifungal, anti-inflammatory, and immunomodulatory effects [11,13,14]. Pontoppidan et al. (2015) reported that colostrum reduces the toxic effects of busulfan and cyclophosphamide on the intestine [38]. Shen et al. (2016) reported the beneficial results of colostrum in doxorubicin-induced intestinal toxicity [39]. Karabacak et al. (2018) stated that paracetamol-induced tissue damage was reduced with colostrum [40]. Abdelmeguid et al. (2021) noted that bovine colostrum prevents hepatotoxicity caused by polycyclic aromatic hydrocarbons [41]. Celebi et al. reported that bovine colostrum has no significant effect on healing in 5-FU-induced oral mucositis [16]. In the literature review, no study addressed the impact of BC on liver, kidney, and heart tissue damage due to 5-FU use. In this study, it was seen that BC protected by reducing inflammation, oxidative stress, apoptosis, and DNA damage in liver, kidney, and heart tissue caused by 5-FU, although the statistical analysis of the score did not reveal any significance.

## 5. Conclusions

This study investigated the protective effect of BC against the striking side effects of 5-FU, such as severe pathological lesions in the liver, kidney, and heart tissue. In addition, it was determined that inflammation, oxidative stress, apoptosis, and DNA damage occurred in these organs. It was seen that the use of BC reversed these effects. As a result of this study, it was seen that it provided a protective effect against liver, kidney, and heart toxicities caused by 5-FU. Although more studies are needed, it is hoped that BC will improve prognosis by both reducing the side effects of 5-FU, a good chemotherapeutic agent, and its antineoplastic properties.

## Data Availability

The data presented in this study are available upon request from the corresponding authors.

## Abbreviations

The following abbreviations are used in this manuscript:

5-FU	5-fluorouracil
BC	Bovine colostrum
dTMP	Deoxythymidine monophosphate
NO	Nitric oxide
Ig	Immunoglobulin
IGF	Insulin-like growth factor
EGF	Epidermal growth factor
TGF	Transforming growth factor
VEGF	Vascular endothelial growth factor
PDGF	Platelet-derived growth factor
DNLs	Degenerative-necrotic lesions
ICIs	Inflammatory cell infiltrates
VCs	Vascular changes
